# Murine allele and transgene symbols: ensuring unique, concise, and informative nomenclature

**DOI:** 10.1007/s00335-021-09902-3

**Published:** 2021-08-14

**Authors:** M. N. Perry, C. L. Smith

**Affiliations:** Mouse Genome Informatics (MGI), Jackcon Laboratory, Bar Harbor, ME USA

## Abstract

In addition to naturally occurring sequence variation and spontaneous mutations, a wide array of technologies exist for modifying the mouse genome. Standardized nomenclature, including allele, transgene, and other mutation nomenclature, as well as persistent unique identifiers (PUID) are critical for effective scientific communication, comparison of results, and integration of data into knowledgebases such as Mouse Genome Informatics (MGI), Alliance for Genome Resources, and International Mouse Strain Resource (IMSR). As well as being the authoritative source for mouse gene, allele, and strain nomenclature, MGI integrates published and unpublished genomic, phenotypic, and expression data while linking to other online resources for a complete view of the mouse as a valuable model organism. The International Committee on Standardized Genetic Nomenclature for Mice has developed allele nomenclature rules and guidelines that take into account the number of genes impacted, the method of allele generation, and the nature of the sequence alteration. To capture details that cannot be included in allele symbols, MGI has further developed allele to gene relationships using sequence ontology (SO) definitions for mutations that provide links between alleles and the genes affected. MGI is also using (HGVS) variant nomenclature for variants associated with alleles that will enhance searching for mutations and will improve cross-species comparison. With the ability to assign unique and informative symbols as well as to link alleles with more than one gene, allele and transgene nomenclature rules and guidelines provide an unambiguous way to represent alterations in the mouse genome and facilitate data integration among multiple resources such the Alliance of Genome Resources and International Mouse Strain Resource.

**Supplementary Information:**

The online version contains supplementary material available at 10.1007/s00335-021-09902-3.

## Introduction

Reproducibility and communication of scientific results relies on precise methods and traceable materials. When those materials include biological entities such as genes, alleles, and organisms, additional care must be taken to ensure that these biological units are identified by unique and persistent identifiers (PUI) such as standardized nomenclature and accession identifiers (IDs). Allele and transgene nomenclature creates a standardized symbolic language that must balance specificity, stability, uniqueness, and informativeness while adhering to a standard set of unifying rules and reasonable character length. The International Committee on Standardized Genetic Nomenclature for Mice has established rules and guidelines that continue to evolve as new technologies are invented to manipulate the genome and researchers develop ever more complex alleles. The full rules and guidelines for the nomenclature of mouse genes, alleles, and strains are available at the Mouse Nomenclature Home Page (http://www.informatics.jax.org/mgihome/nomen/index.shtml).

**Fig. 1:**
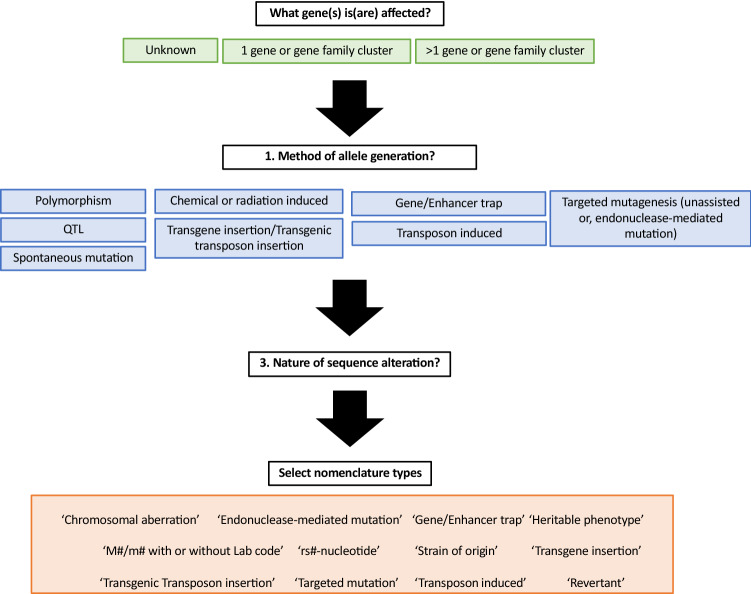
Selecting the appropriate allele or transgene nomenclature style requires the consideration of three factors

While murine gene symbols follow the established human symbols, allele nomenclature for mouse and rat is based on a series of requirements about the information a symbol should convey including the gene(s) altered, the method of generation, and the nature of the alteration (Fig. 1). By parsing this information and combining it with serial numbers and ILAR-registered laboratory codes (https://www.nationalacademies.org/ilar/lab-code-database), unique, concise, and informative allele and transgene symbols can be generated and registered with Mouse Genome Informatics (MGI), the foremost resource for mouse genetic information.

Stability is a key requirement for nomenclature. Changes to the guidelines must be approved by International Committee on Standardized Genetic Nomenclature for Mice and in some instances symbols have been grandfathered in to preserve the symbols for long-standing mouse mutations where the symbol has been widely used in the literature and is recognized by the scientific community, even if the symbol no longer conforms to updated nomenclature rules and guidelines. When symbols are changed, MGI includes the old symbol as a synonym. On rare occasions, MGI has adopted long-standing and widely recognized symbols for highly modified, non-mouse markers such as CAG (a ubiquitous promoter consisting of a fusion of the cytomegalovirus (CMV) enhancer and chicken beta-actin promoter; Alexopoulou et al. 2008). However, effort must be made to ensure that the symbols used in allele nomenclature are unique and only contain gene symbols registered at other authoritative nomenclature resources.

Although some allele symbols may change as more become known about the alleles, MGI assigns a unique accession identifier to these and most other data objects within the knowledgebase. By specifying the MGI-registered allele symbol and the accession ID in publications, researchers can be assured of utmost clarity in their data presentation and provide the scientific community basic information to facilitate reproducibility of results. The use of the Sequence Ontology (SO; http://www.sequenceontology.org/; Eilbeck 2005) to develop relationships to link individual alleles to multiple genes with standardized mutation terms provides additional and reciprocal data links not captured in the symbols. Sequence variant nomenclature and standardized numeric identifiers, such as repository IDs, provide additional reference points for cross-species comparison at MGI and the Alliance for Genome Resources and to retrieve alleles of interest and the mouse strains bearing them using the International Mouse Strain Resource (IMSR; described below; Alliance of Genome Resources Consortium 2020; Eppig et al. 2015). In addition to providing long-term recognition of the creator of a biological resource, the use of unique and persistent identifiers such as standardized nomenclature and accession IDs facilitates reproducibility and integration of scientific data across platforms and between organisms.

## Methods of generation-based nomenclature

The principal methods of allele generation in mice include spontaneous; induced by chemical or radiation treatment; transgenic insertion (including transgenic transposon insertion); gene/enhancer trapped; transposon induced; and targeted (homologous recombination, endonuclease-mediated). Each method of allele generation has a specific requirement for how to construct an allele symbol including requirement for a gene symbol, method of generation prefix, serial number, and ILAR-registered laboratory code (detailed below and in Table 1). These method-specific prefixes are primarily used for alleles of a single gene, whereas mutations that impact more than one gene or gene family cluster—a set of related genes located in an uninterrupted genetic intervals such as the Hoxa genes (see below)—utilize heritable phenotypic allele and chromosomal aberration nomenclature regardless of allele generation method. Specific nomenclature is also used to designate modifications of existing alleles by in vivo recombination or resulting in reversion of the mutation allele to the wild-type allele regardless of method of generation.

**Table 1:** Standard allele nomenclature types by method of generation or identification

Generation	Heritable phenotype symbol	Gene	Allele symbol				Examples
			Mutation abbreviation	Parenthesis	Serial number	ILAR laboratory code	
Chemical and/or radiation induce	Optional	Optional	m or M (optional)	None	Required	Optional	*Map3k4*^*byg*^ *Alx4*^*M1Yzcm*^ *Aqp2*^*F204V*^
Chromosomal aberration		None	See Table 2	chr#, boundaries	Required	Required	Del(3Bglap2-Bglap)1Vari
Endonuclease-mediated mutation (CRISPR, ZFN, TALEN)		Required	em	See Table 3	Required	Required	*Ace2*^*em1Smoc*^
Gene/enhancer trap		Optional	Gt, Et	Mutant cell line or vector	Mutant cell line or vector	Required	Et(cre/ERT2)13866Rdav *Bcor*^*Gt(XE541)Byg*^ *C7*^*Gt1Tigm*^
QTL	Required	Optional	None	None	Optional	None	*Cafq1*^*APN*^
Revertant	Mutation reverted	Required	Use that of reverting mutation	None	Required	Required	a^a+em1Tk^ Hr^hr+^
Spontaneous mutation (parental allele unknown)	Optional	Optional	None	None	Optional	None	*Bag3r*^*s31544129-G*^ *Ctse*^*129S/SvHsd*^ *Usf1*^*soc*^
Spontaneous mutation (parental allele unknown)	Optional	Optional	m or M (optional)	None	Optional	Optional	*Apoa1*^*m1Pgrs*^ *Clic5*^*jbg*^ *Cm* *Fpr3*^*del*^ *Itpr3*^*C57BL/6 J*^
Targeted mutation via un-assisted homologous recombination		Required	tm	See Table 3	Required	Required	*Hprt*^*tm1(CAG-mCherry/Villin)Syr*^ *Il4ra*^*tm1Fbb*^
Transgene		None	Tg	Promoter or enhancer plus all expressed sequences	Required	Required	Tg(BEST1-rtTA,tetO-cre)1Yzl
Transgenic transposon insertion		None	TgTn	Transposase sites, promoter and expression cassette or construct name	Required	Required	TgTn(itol2-CAG-GFP)1Dla TgTn(mm-DTT)1Ddra
Transposon induced		Optional	Tn	Transposase, transposon concatemer	Required	Required	Tn(sb-SBlac)15.179039Fsp *Mdc1*^*Tn(pb-ZG-s)1.1Mrc*^ *Nmnat2*^*Tn(sb-Tyr)2172.P9KK4BOve*^

### What gene is mutated?

The first criteria for deciding the type of allele symbol to use is the gene or genes affected, if known. Mutations that are identified only by their phenotype, including quantitative trait loci and uncloned spontaneous or induced phenotypic mutations, are represented by heritable phenotypic symbols. Mutations that involve more than one gene or gene family cluster are assigned chromosomal aberration nomenclature regardless of generation method. MGI assigns markers symbols to represent defined gene clusters and regions in the genome. A gene family cluster is a set of related genes in the same genomic segment interrupted by, at most, a few other genes. For example, the homeobox A cluster (Hoxa; supplemental Table 1 contains the MGI accession IDs for all genes and alleles referenced in this publication) is located between 52155590 and 52260880 on chromosome 6 (GRCm38) and contains eleven Hoxa gene family members (*Hoxa1-7, Hoxa9-11, and Hoxa13*). Mutations that affect more than one member of the cluster can be assigned to the cluster marker and are treated similar to single gene mutations whose nomenclature is prescribed by the generation type (see below).

### Heritable phenotypic mutation symbols

Heritable phenotypic mutations that are not cloned are initially assigned nomenclature based on the phenotype or the center that generated the mutation. Such mutations can arise spontaneously or may be induced, such as by radiation (e.g., gamma irradiation) or a chemical (e.g., N-ethyl-N-nitrosourea, ENU). Heritable phenotype marker (SO:0001500) symbols are typically up to five letters in length with the first letter capital for dominant and semi-dominant phenotypes and the first letter lower case for recessive phenotypes (e.g., *Cm*; Xue et al. 1999). Mutations that are first identified by their phenotype then later mapped to a single gene or gene family clusters will have their symbols updated to place the heritable phenotypic symbol as a allele symbol of the gene (e.g., *Enpp1*^*ttw*^, *Gli3*^*Xt*^; Hosoda et al. 1981; Lyon et al. 1964; Pohl et al. 1990). When a heritable phenotypic marker is found to be a chromosomal aberration, the heritable phenotypic marker may be retained or replaced with the appropriate chromosomal aberration marker. Conversely, chromosomal aberrations are represented by a heritable phenotypic symbol (e.g., Mp) when chromosomal aberration nomenclature cannot be used due to the complexity of the mutation (Rainger et al. 2013).

### QTL

Quantitative trait loci (QTL) are genomic segments associated with measurable traits that differ between two populations. These regions can be mapped to a single gene or span multigenic intervals megabases in length. They are often named based on the trait being examined (e.g., *Cafq1*—caffeine metabolism QTL 1) with the allele designations represented as the study populations (e.g., *Cafq1*^*APN*^ and *Cafq1*^*C3H/HeJ*^; Casley et al. 1999). Occasionally, the genes responsible for the differences in the trait expression can be isolated to a single gene. However, often multiple sequence differences may contribute to the strain-specific phenotype. While a corresponding mutation or mutations within a single gene may be identified in the QTL interval, these mutations may not fully represent the sequence variations that account for all of the trait captured by the QTL. As such, a separate allele record is created for the spontaneous mutation and an allele to marker relationship ‘mutation involves’ to associate the spontaneous mutation allele and any other sequence variants identified with the QTL marker.

### Chromosomal aberrations

Regardless of allele generation method, mutations that alter more than one gene or gene family cluster are denoted using chromosomal aberration nomenclature. These chromosomal aberrations capture deletions, duplications, inversions, insertions, Robertsonian translocations, translocations, transchromosomal insertions (insertion of a chromosome segment from another species), and trisomies. The chromosomal aberration symbol contains a prefix specifying the type of rearrangement (see Table 2), parenthesis containing the chromosome(s) altered, and genes comprising the boundaries of the affected genetic segment if it is known, serial number, and laboratory code. The serial number is sequential for the laboratory for each type of chromosomal aberration (e.g., Dp(7)1H, Del(10)1H, Del(10)2H, Is(In;5)1H, Is(1;11)2H)—a change from earlier numbering schemes that required a unique serial number for each chromosomal aberration from a given laboratory. Elaboration of describing the breakpoints of the altered segment using bands can be found on the International Mouse Nomenclature Committee website (http://www.informatics.jax.org/mgihome/nomen/index.shtml under ‘Rules for Nomenclature of Chromosome Aberrations’).

**Table 2:** Multi-gene (not within a gene family cluster) allele nomenclature regardless of method of generation

Aberration	Abbrev.	Chromosome	Examples
Deletion	Del	Chromosome, boundaries, serial number, laboratory code	Del(3Bglap2-Bglap)1Vari
Duplication	Dp	Same as above	Dp(16Cbr1-Fam3b)1Rhr
Inversion	In	Same as above	In(7Oca2;7Sox6)100H
Insertion	Is	Same as above	Is(17;In2)1Gso
Robertsonian translocation	Rb	Chromosomes, serial number, laboratory code	Rb(1.11)2Mpl
Translocation	T	Chromosomes, serial number, laboratory code	T(X;16)16H
Transchromosomal	Tc	Species, chromosomes, serial number, laboratory code	Tc(HSA14)SC20Ktom
Trisomy	Ts	Chromosome(s) affected with the translocated chromosome in superscript when different from insertion chromosome	Ts(17^16^)65Dn

All symbols require an aberration abbreviation, chromosome(s), serial number, and ILAR-registered laboratory code

Since chromosomal aberration symbols only contain the boundaries of the mutation, MGI uses the ‘mutation involves’ relationship (see below) to link all the genes affected by the sequence alteration to the chromosomal aberration symbol. For example, Del(3Bglap2-Bglap)1Vari, the first multigenic deletion produced in Dr. Bart William’s laboratory encompassing bone gamma-carboxyglutamate protein 2 through bone gamma-carboxyglutamate protein on Chr 3, and is linked in MGI through the ‘mutation involves’ relationship to the two bone gamma-carboxyglutamate proteins and the predicted gene contained within the interval (Gm6821; Diegel et al. 2020).

### What method of generation for single gene or gene family cluster alleles?

Mutations in individual genes or within a gene family cluster can be generated through a number of methods. They may arise spontaneously, but can also be randomly induced by chemical or radiation treatment. Random transgenic insertion (including transgenes, traps, and transposons) can introduce expressed exogenous sequences as well as disrupt endogenous gene expression or co-opt regulatory function by virtue of the insertion event, insertion of splice acceptors, or mobilization of an inserted transposon concatemer. Mutagenesis of specific genes and genomic regions is achieved through homologous recombination and endonuclease-mediated non-homologous end joining and homologous recombination with a donor plasmid. Each method of generation has specific requirement for what to include in the allele symbol (detailed below and in Table 1).

### Spontaneous mutations (strain variants and reference SNP, mutation serial number)

The mutations that arise spontaneously in strains range from single nucleotide variants to large chromosomal rearrangements. When strain variants are identified without one identified as the parental allele that underwent mutation, the alleles are referred by different symbols depending on the information available and encompasses heritable phenotypic alleles, strain-specific alleles symbols, and reference SNP (rs) nomenclature.

Spontaneous mutations are initially identified by phenotype or sequence. Phenotypic mutations are generally assigned a phenotypic name and corresponding symbol. Once the gene is identified that symbol becomes a superscript of the gene for the full allele symbol, unless the underlying molecular lesion is multigenic or in an intragenic region. Spontaneous mutations also underlie genetic drift and there are nomenclature rules for assigning alleles to sequence variations between strains. If a mutation is unique to a particular inbred strain then it is permissible to name that allele by the strain name, such as *Nnt*^*C57BL/6J*^ (Toye et al. 2005), understanding that this term refers to the entire gene sequence. More frequently a mutation found in one inbred strain is shared by several others, having been fixed in a shared ancestral genome. In the absence of a phenotypic name, these mutations are named simply with an m and a serial number for the gene impacted, such as *Il2*^*m1*^ (Choi et al. 2002), which is found in MRL/MpJ, SJL/J, and NOD/ShiLitJ, or *Ogg1*^*m2*^, which is found in NZB/N, NFS, and SLJ/J. When it is not known whether a specific sequence variation is specific to one strain or may be in others, if closely related strains have not been assessed, then the m# nomenclature is a more inclusive nomenclature (Choi et al. 1999).

Spontaneous mutations may arise as deviants in a subline and are not a characteristic of the parental strain. Instead of being assigned a phenotypic name and symbol, these mutations can also be represented by the more general mutation prefix m (lowercase) for recessive mutations or M (uppercase) for dominant and semi-dominant mutations, followed by a serial number, and an optional laboratory code when the mutation is known to have arisen in a specific laboratory (e.g., *Apoa1*^*m1Pgrs*^; Wiltshire et al 2012). It is not recommended to use the amino acid substitution as amino acid numbering can differ in multiple transcripts of the same gene. For example, *Tyk2*^*E775K*^ describes a point mutation whose numbering has changed over time and varies depending on the transcript (Tyk2:NM_001205312.1:c.2404G > A:p.(Glu802Lys), Tyk2:NM_018793.2:c.2335G > A:p.(Glu779Lys), Tyk2:XM_006510492.3:c.2023G > A:p.(Glu675Lys), Tyk2:XM_006510493.2:c.1846G > A:p.(Glu616Lys), Tyk2:XM_011242579.2:c.2335G > A:p.(Glu779Lys), Tyk2:XR_001778942.1:n.2510G > A) (Shaw et al. 2003).

The European Variation Archive (https://www.ebi.ac.uk/eva/; Cook et al. 2016) provides a catalog of mouse sequence polymorphisms with registered reference SNP (rs) identifiers and replaces NCBI’s dbSNP and dbVar for non-primate SNPs. These rs designations can be used to refer to the variant. When the rs variant is present in a known gene, that allele is represented by the rs number in superscript as the allele symbol for the gene symbol with the nucleotide specified after a hyphen(e.g., *Bag3*^*rs31544129−G*^; McClung et al. 2017). The rs designations should be registered with EVA prior to assignment in official allele nomenclature. MGI offers a mouse-specific SNP query form in addition to those offered at EVA and the Mouse Genome Project (https://www.sanger.ac.uk/sanger/Mouse_SnpViewer/rel-1505; Keane et al. 2011; Yalcin et al. 2011).

Multigenic spontaneous mutations that alter more than one gene or gene family cluster are represented by chromosomal aberration nomenclature (Del(5Kit-Cep135)1Utr; Mizuno et al. 2015).

### Randomly induced mutations (chemical or radiation induced)

By making use of DNA intercalating chemicals and/or radiation exposure, researchers generated the first cataloged induced mutations ranging from point mutations to large chromosomal aberrations (Flaherty et al. 1998; Gondo et al. 2010). The allele symbol nomenclature for these depends on whether the mutation has been molecularly characterized and if it affects one or more genes.

While heritable phenotypic allele symbols are allowed (e.g., *zoef*, *Adgrv1*^*rueda*^; Schwander et al. 2007; Wansleeben et al. 2011), the mutation serial number with the laboratory code where the mutations occurred (e.g., *Tomt*^*m1Btlr*^; Du et al. 2008), or rs number (e.g., *Bag3*^*rs31544129−G*^) is more informative (McClung et al. 2017). The use of allele symbols with amino acid substitution designations is discouraged as mentioned above since it can cause confusion between protein products from alternate transcripts that may have different amino acid numbering.

### Transgene insertion and transgenic transposon insertion

Whereas chemical- and radiation-induced mutations make point mutations or remove, duplicate, or rearrange endogenous genomic material, the insertion of exogenous genetic material has been achieved through random transgenic insertion to insert expression units, including transposon concatemers. The random nature of this integration has the potential for positional effects on expression as well as disruption of the integration site function.

The ability to randomly insert constructs of various length to achieve expression in mice has been a powerful tool for many decades. These random insertions are represented with transgene insertion nomenclature using the prefixes Tg. All transgenes require a line number and laboratory code. It is crucial that each line number represent a unique founder line that has achieved germline transmission to register the transgene symbol with MGI. Due differences in insertion site, the expression profile and phenotypic spectrum may vary between lines. The parenthesis can contain either the BAC number or the promoter(s) and enhancer(s) followed by a hyphen and the expressed sequences (e.g., Tg(BEST1-rtTA,tetO-cre)1Yzl; Ueki et al. 2009).

The specific subsets of transgene insertions that are transgenic transposons require a distinct prefix (TgTn), line number or name, and laboratory code. Transgenic transposon are transgenes which carry a series of transposon constructs (concatemer) to be later mobilized. Within the parenthesis of transgenic transposons, the transposase symbol is followed by a hyphen and either the transposon construct name or contents (e.g., TgTn(itol2-CAG-GFP)1Dla, TgTn(mm-DTT)1Ddra; de Wit et al. 2010; Keng et al. 2009).

### Gene/enhancer traps, transposon-induced mutations

Gene and enhancer traps are constructs that contain splice acceptor and/or minimal promoters and a reporter designed to subvert the regulatory regions of a gene or DNA segment to drive expression of a reporter gene rather than the endogenous transcript. Gene traps can both achieve gene-specific expression of a reporter gene and produce a null or hypomorphic allele depending on the design. Enhancer traps also contain a minimal promoter to capture the expression patterns of enhancers. Because of the ability of these traps to randomly integrate into the genome, their insertion sites may be difficult to map or fall within intergenic segments (Abuin et al. 2007). As such, they do not require a gene designation for the enhancer or gene trap symbol. Traps that map to a single gene or gene family cluster are represented by a superscript containing the trap symbol. These symbols contain a prefix (Et or Gt), optional parenthesis containing the mutant cell line or vector, a line number when no parenthesis are present, and a laboratory code (e.g., Bcor^Gt(XE541)Byg^, C7^Gt1Tigm^, Cdk1^Gt(pGT1−3)1Bbd^, Et(cre/ERT2)13866Rdav; Cox et al. 2010; Davis 2009; Santamaría et al. 2007; Welsh et al. 2012).

The power of transposon-induced mutagenesis is the ability to mobilize and remobilize transposable elements introduced using a transgenic transposon. In addition to the transposon prefix (Tn), transposase long terminal repeats (e.g., sb—sleeping beauty, pg—piggyback), vector name, line number, and laboratory code, nomenclature also provides a link between the original transgenic transposon concatemer and subsequent mobilizations using the decimal and chains of line numbers whether the mobilized transposon insertion occurs within a gene or an intergenic region (e.g., Tn(sb-SBlac)15.179039Fsp, *Mdc1*^*Tn(pb−ZG−s)1.1Mrc*^; Ruf et al. 2011; Wu et al 2007). For example, when the transgenic transposon TgTn(sb-Tyr)2172Ove is mobilized with the sleeping beauty transposase, line P9KK4B contains integration into nicotinamide nucleotide adenylyltransferase 2 gene (*Nmnat2*^*Tn(sb−Tyr)2172.P9KK4BOve*^; Hicks et al. 2012). Thus, nomenclature can be harnessed to inform the method of generation, gene affected, and source laboratory as well as relate alleles to the common transgenic transposon.

### Targeted mutations (homologous recombination and endonuclease-mediated)

Despite the wide array of mutant alleles that have been generated by spontaneous and induced mutagenesis, the development of technologies to target specific genomic sequences and either remove or add sequences necessitated an expansion of allele nomenclature. Researchers continue to produce increasingly elegant targeting mutations that alter gene function, create molecular tools, and recapitulate human diseases with more specificity than ever before. Targeted mutations are subdivided between homologous recombination and endonuclease assisted with specific nomenclature guidelines governing inclusion of exogenous sequence symbols. The allele symbol in superscript is not unique and requires reference to the targeted gene unless more than on continuous sequence is altered, in which case, chromosomal aberration is utilized.

Homologous recombination in ES cells has been the principal method for generating targeted mutations for decades. The symbol begins with the mutated gene or gene family cluster symbol and the allele superscript contains the prefix tm, a serial number representing the number of alleles this laboratory has made in this gene, and an ILAR-registered laboratory code (e.g., *Il4ra*^*tm1Fbb*^; Mohrs et al., 1999). If expressed exogenous sequence is inserted into the gene then that information may be represented in parenthesis before the laboratory code. Additionally, the inclusion of the parenthesis is only warranted to capture certain additional types of information (Table 3 and elaborated on below; e.g., *Hprt*^*tm1(CAG−mCherry/Villin)Syr*^; Hsiao et al., 2011). It is tempting to try to include exon numbering or non-standard abbreviations in the symbol; however, they are often not standardized and subject to change. Hence, MGI associates nicknames used in publication to the official symbol. As with alleles generated through other methods, targeted mutation alleles can be associated with more than one gene using the relationships established in MGI, which eases the burden of trying to fit too much information into the allele symbol and provides critical data links between alleles and genes or other mutations. For example, the expresses component relationship links the allele record for *Actb*^*tm3.1(Sirt1)Npa*^ to the gene record for mouse *Sirt1* (Bordone et al. 2007). Through this relationship the phenotype generated through the expression *Sirt1* is associated with *Sirt1* and not *Actb* (Bello et al. 2015).

**Table 3:** Targeted (traditional targeted or endonuclease-mediated mutations) knock-in allele parenthesis nomenclature

	Do not include	Include	Examples
Inserted expressed sequence			
Expressed protein coding genes		Only non-endogenous sequences or those from another species	*Ctla4*^*tm1.1(CTLA4)Geno*^ *Kcna5*^*tm1(Kcna1)Lndn*^
Isoform		Only for exogenous inserted sequence	*Apoe*^*tm1(APOE_i4)Sfu*^
microRNA sponge	If the source of a binding sequence is not available and/or there is no unique symbol for it	If source sequence is known	*Gt(ROSA)26Sor*^*tm2Jake*^
Modified genes	Point mutation of the endogenous gene	* (asterisk), up to 3 point mutations for exogenous genes	*Krt12*^tm1.1(KRT12*L132P)Arte^
RNAi constructs		RNAi: target	*Col1a1*^*tm1(tetO−RNAi:Rps19)Karl*^
Reporters		Only reporters driven by an exogenous promoter or fused to an inserted expressed sequence	*Nfatc1*^*em2(GFP/cre)Bzsh*^ *Igs2*^*em2(CAG−tdTomato)Gpt*^
Inserted not expressed sequence			
	Epitope tag		
	Localization sequences		
	Point mutations		
	Recombinase sites		
	Selection gene		
	Spacer sequence		
	STOP sequence		

Since endonuclease-mediated mutation, such as the CRISPR/Cas system, zinc finger endonucleases (ZFN), and transcription activator-like effector nucleases (TALEN), differ from traditional targeting in their ability to generate both random and prescribed genomic alterations at target sites, the international nomenclature committee has adopted the prefix ‘em’ for endonuclease-mediated mutation (e.g., *Ace2*^*em1Smoc*^; https://www.modelorg.com/en/portal/article/index/id/5288/post_type/3.html). Beyond the prefix, endonuclease-mediated mutation alleles follow the same guidelines as traditional targeted mutation in requiring a serial number and laboratory code with the optional parenthesis to summarize inserted expressed sequences with restrictions on content (detailed below). Although several founders with a variety of mutations can be produced from the same RNA guides, MGI treats each unique mutation in a different genetic background as a new serial number for the laboratory.

### Knock-in parenthesis content

A source of confusion in targeted nomenclature, whether generated by traditional gene targeting through homologous recombination or endonuclease-mediated mutation, is what to include in the optional parenthesis. While the parenthesis provides a place to capture additional information, it is not a free text field (Table 3). The contents are restricted to authoritative gene symbols of inserted expressed sequences either exogenous to the location of insertion or from another species, commercial reporter gene symbols driven by exogenous promoters (the exogenous promoter separated from the expressed sequence with a hyphen) or fused to an inserted expressed sequence (fusion indicated by a forward slash), RNAi-targeting constructs, and mutations of inserted expressed exogenous genes (denoted by an asterisks with up to three amino acid substitutions; Table 2). Not included in the parenthesis are reporter genes driven by the endogenous promoter and not fused, microRNA sponges if the source of a binding sequence is not available and/or there is no unique symbol for it, endogenous gene modifications other than fusion to an exogenous inserted sequence, recombinase sites, translation stop sequences, selection cassettes, spacer sequences, epitope tags, etc. While the parenthesis can expand the information contained within an allele symbol, there is a limit to how long they can be—the gene and allele symbol cannot be more than fifty characters in length—and what they contain to maximize information and maintain consistency between symbols.

### Modification of mutant alleles (recombination and revertant)

Mutant alleles are subject to changes whether spontaneously or in a more targeted manner that either further alter the allele or revert it to a wild-type allele (revertant) or are subject to recombinase-mediated recombination to removed or insert sequence.

Recombinase-mediated events that remove a portion of the originally targeted vector in vivo, often called derivative alleles, are denoted with a decimal and serial number (e.g., *Pou5f1*^*tm1.1Scho*^), while recombinase-mediated cassette exchange (RMCE) which introduces new sequence into the allele is considered a novel allele, not a derivative allele, and, therefore, is assigned the next serial number and laboratory code for the laboratory that generated it (e.g., *Col1a1*^*tm2(tetO−Ccnb2)Jvd*^ generated through recombination of *Col1a1*^tm13(neo/hygro*)Jae^) (Hochedlinger et al. 2005; Kehler et al. 2004; Nam and van Deursen 2014). When the gene trap construct contains a recombinase site flanked elements, the allele produced through recombination is designated with a decimal (e.g., *Nipbl*^*Gt(EUCE313f02)1.1Hmgu*^) similar to recombination of targeted mutations (Santos et al. 2016).

Whether spontaneous or engineered, mutations can occur in mutant alleles that fully restores the wild-type sequence or phenotype. These revertant alleles are represented by an allele symbol that contains the original mutation, a ‘ + ’ character, and, if engineered, the allele symbol of the engineered revertant mutation (e.g., *Hr*^*hr*+^, *a*^*a*+*em1Tk*^; Stoye et al. 1988; Tanave et al. 2019). If targeted mutation restores the original mutation but adds additional sequence, then revertant nomenclature should not be used. These alleles engineered in the context of an existing mutation are designated according to their mutation generation type and the original mutation represented in the molecular note and/or the strain of origin. MGI is developing a relationship to link alleles to the alleles they are generated from and vice versa.

## Allele to gene relationships

As much information as nomenclature attempts to capture, there is a limit to what can be included without making symbols excessively long, convoluted, and inconsistent between different allele symbols. Because of this limitation, MGI has developed a set of data relationships that link alleles and transgenes to multiple genes (Table 4). The allele-specific relationships used in MGI were developed in accordance with the mutation definitions found in the SO and include ‘decreased translation product level’ for RNAi target genes, ‘expresses component’ for exogenous expressed mouse or mouse orthologous sequences, and ‘mutation involves’ for additional gene sequences altered by the allele through deletion, duplication, inversion, or unaltered but contained within the interval. These relationships provide stable integration of alleles with their related genes allowing improved access to alleles and transgenes with common elements without adding additional complexity to allele and transgene symbols in addition to facilitating links between mouse transgenes and knock-in alleles to their expressed human genes on multi-model organism web portals like the Alliance of Genome Resources (discussed further below).

**Table 4:** SO-based allele to gene relationships used in MGI

Relationship	SO term	Allele	Altered gene
Decreased translation product level	SO:0001555	*Col1a1*^*tm1(tetO−RNAi:Rps19)Karl*^	*Rps19*
Expresses component		*Kcna5*^*tm1(Kcna1)Lndn*^	*Kcna1*
Mutation involves: • Deletion • Duplication • Inversion • Gene_fusion	• SO:0000159 • SO:1000035 • SO:1000036 • SO:0001565	• Del(3Bglap2-Bglap)1Vari • Dp(Y)1H • *Ppcd1* • *Wld*^*s*^	• *Balap2*, *Balap2*, *Gm6821* • *Sry*, *Zfy2*, *Kdm5d* • *Dzank1* • *Nmnat1*, *Ube4b*

## Additional identifiers, and resources (HVGS, alliance, RRID, IMSR)

In addition to providing official allele nomenclature and phenotypic data associated with published and unpublished alleles, MGI is cataloging variant nomenclature for alleles using the standard established by the human variant genome sequence (HVGS; https://varnomen.hgvs.org/; Laros et al, 2011). HVGS offers a standardized format for capturing the alteration in genomic, transcript, and protein sequences from single nucleotide changes to more complicated rearrangement. For example, Adam17:NM_001277266.1:c.851C > T:p.(Thr284Met) is but one of the numerous HGVS designations that describe the single point mutation in the spontaneous mutation ‘waved with open eyelids’ that occurs within the gene ‘a disintegrin and metallopeptidase domain 7’ (*Adam17*^*woe*^) at the level of genome transcript and protein (see Table 5; Hassemer et al. 2010). These designations with sequence reference provide unambiguous context for the numbering the nucleotide and resulting protein change. In the near future, MGI plans to present searchable HVGS variant description for alleles containing simple nucleotide changes and small deletions.

**Table 5:** HGVS variant nomenclature describing the point mutation in *Adam17*^*woe*^ at the genome, transcript, and protein levels from the Alliance for Genome Resources (https://www.alliancegenome.org/allele/MGI:3625359)

HGVS.g Name	HGVS.c Name	HGVS.p Name
(GRCm38)12:21345670G > A	ENSEMBL:ENSMUST00000064536.1:c.794C > T	ENSEMBL:ENSMUSP00000067953.1:p.Thr265Met
12:g.21345670G > A	ENSEMBL:ENSMUST00000101551.1:c.851C > T	ENSEMBL:ENSMUSP00000099087.1:p.Thr284Met
NC_000078.6:g.21345670G > A	ENSEMBL:ENSMUST00000127974.1:c.*144C > T	ENSEMBL:ENSMUSP00000136407.1:p.Thr265Met
	ENSEMBL:ENSMUST00000145118.1:c.794C > T	RefSeq:NP_001264195.1:p.Thr284Met
	ENSEMBL:ENSMUST00000232107.1:c.324 + 8250C > T	RefSeq:NP_001278800.1:p.Thr27Met
	ENSEMBL:ENSMUST00000232526.1:c.*370-2693C > T	RefSeq:NP_033745.4:p.Thr265Met
	RefSeq:NM_001277266.1:c.851C > T	RefSeq:XP_030102390.1:p.Thr102Met
	RefSeq:NM_001291871.1:c.80C > T	
	RefSeq:NM_009615.6:c.794C > T	
	RefSeq:XM_030246530.1:c.305C > T	

HVGS variant nomenclature for mouse mutations is also being incorporated at the Alliance of Genome Resources (https://www.alliancegenome.org/). Mouse allele pages at this resource already include mouse variant nomenclature provided by MGI that allows for exact placement of the mutation on the JBrowse platform for genome visualization and integration of biological data (https://jbrowse.org; Buels et al. 2016). These data are available for download from this resource.

The International Mouse Strain Resource (IMSR; http://www.findmice.org) offers a web portal to assess information and direct weblinks to mouse strains held by twenty-nine repositories from all over the world. As of March 2021, IMSR contains mouse strain listings available in the following states: 217,419 ES cells; 42,582 sperm; 17,427 embryos; 20,089 archived; 8,300 live; and 258 ovaries. All strains submitted to IMSR are periodically reviewed by MGI and assigned official gene, allele, and strain symbols to facilitate integration of mouse strain information.

Resource identifier (RRIDs) are often used in publications and elsewhere to refer to specific resources such as antibodies, plasmids, cell lines, tools, and model organisms. These unique identifiers are available to search at SciCrunch (https://scicrunch.org) and pre-pend RRID: to a resource ID and resource provided identification code. By mining a number of research resources, SciCrunch has developed an interface to search for multiple resource types. SciCrunch appends RRID to public MGI genotype and strain IDs as well as strains listed in IMSR. While strains from MGI receive the prefix RRID:MGI:, the strains from IMSR append the repository ID prefix (e.g., RRID:IMSR_EM:10306 for STOCK *Lipa*^*tm1a(EUCOMM)Hmgu*^/Biat). RRIDs are useful for defined biological and chemical entities. However, the most specific identifier for a mouse allele independent of its background strain is the MGI allele ID.

## Conclusion

Unique, concise, and informative gene and allele nomenclature is key to scientific communication, data integration, and reproducibility of results. There is an ever-evolving debate over stability versus adaptability and inclusiveness or simplicity that must be struck to make a robust symbolic language of allele nomenclature that is meaningful and useful to the scientific community. The International Committee on Standardized Genetic Nomenclature for Mice and MGI, and others strive to work with researchers in naming their alleles and transgene in a manner that best serves the research community and repository resources. MGI offers assistance in naming and registering alleles and transgenes symbols through the nomenclature coordinator (http://www.nomen@jax.org), MGI user support (http://www.informatics.jax.org/mgihome/support/mgi_inbox.shtml), and direct data submissions (http://www.informatics.jax.org/submit.shtml).

## Supplementary Information

Below is the link to the electronic supplementary material.

Supplementary file1 (DOCX 16 kb)
