# Supplementary material for: Murine allele and transgene symbols: ensuring unique, concise, and informative nomenclature
Source: Mamm Genome. 2021 Aug 14;33(1):108–19. doi: 10.1007/s00335-021-09902-3 (PMC8913455; doi:10.1007/s00335-021-09902-3)
Supplement: Supplementary file 1 — Supplementary file1 (DOCX 16 kb) [file 335_2021_9902_MOESM1_ESM.docx]

Supplemental Table 1. MGI symbols, symbol types, method of allele generation, and accession IDs. Not applicable indicates a spontaneous mutation where the parent of origin is not known.

| **Symbol** | **Symbol Type** | **Method of allele generation** | **Accession ID** |
| --- | --- | --- | --- |
| a^a+em1Tk^ | Allele | Revertant (endonuclease-mediated) | MGI:6356874 |
| *Ace2^em1Smoc^* | Allele | Endonuclease-mediated | MGI:6415213 |
| *Actb* | Gene |  | MGI:87904 |
| *Actb^tm3.1(Sirt1)Npa^* | Allele | Targeted (HR) | MGI:4456082 |
| *Adam17^woe^* | Allele | Spontaneous | MGI:3625359 |
| *Adgrv1^rueda^* | Allele | Chemically-induced | MGI:3708270 |
| *Alx4^M1Yzcm^* | Allele | Chemically-induced | MGI:5502864 |
| *Apoa1^m1Pgrs^* | Allele | Spontaneous | MGI:6307002 |
| *Apoe^tm1(APOE_i4)Sfu^* | Allele | Targeted | MGI:2385931 |
| *Aqp2^F204V^* | Allele | Chemically-induced | MGI:3616876 |
| *Bag3^rs31544129-G^* | Allele | Spontaneous | MGI:6197831 |
| *Bcor^Gt(XE541)Byg^* | Allele | Gene trapped | MGI:4126230 |
| *C7^Gt1Tigm^* | Allele | Gene trapped | MGI:6164644 |
| *Cafq1^APN^* | Heritable phenotypic allele | QTL | MGI:2153925 |
| *Cafq1^C3H/HeJ^* | Heritable phenotypic allele | QTL | MGI:2153926 |
| *Cdk1^Gt(pGT1-3)1Bbd^* | Allele | Gene trapped | MGI:3722851 |
| *Clic5^jbg^* | Allele | Spontaneous | MGI:3663254 |
| *Cm* | Heritable phenotypic allele | Radiation induced | MGI:88424 |
| *Col1a1^tm1(tetO-RNAi:Rps19)Karl^* | Allele | Targeted | MGI:5304754 |
| *Col1a1^tm13(neo/hygro*)Jae^* | Allele | Targeted | MGI:5507043 |
| *Col1a1^tm2(tetO-Ccnb2)Jvd^* | Allele | Targeted | MGI:5705291 |
| *Ctla4^tm1.1(CTLA4)Geno^* | Allele | Targeted | MGI:6315564 |
| *Ctse^129S/SvHsd^* | Heritable phenotypic allele | Spontaneous | MGI:5435247 |
| Del(10)1H | Chromosomal aberration | Radiation induced | MGI:104044 |
| Del(10)2H | Chromosomal aberration | Radiation induced | MGI:104045 |
| Del(3Bglap2-Bglap)1Vari | Chromosomal aberration | Endonuclease-mediated | MGI:6256960 |
| Dp(16Cbr1-Fam3b)1Rhr | Chromosomal aberration | Targeted | MGI:3487283 |
| Dp(7)1H | Chromosomal aberration | Radiation induced | MGI:103930 |
| Dp(Y)1H | Chromosomal aberration | Spontaneous | MGI:5471229 |
| *Enpp1^ttw^* | Allele | Spontaneous | MGI:1857079 |
| Et(cre/ERT2)13866Rdav | Enhancer trap insertion | Enhancer trapped | MGI:4947562 |
| *Fpr3^del^* | Allele | Spontaneous | MGI:5800460 |
| *Gli3^Xt^* | Allele | Spontaneous | MGI:1856275 |
| *Gt(ROSA)26Sor^tm2Jake^* | Allele | Targeted | MGI:5474352 |
| *Hoxa* | Cluster |  | MGI:96169 |
| *Hoxa1* | Gene |  | MGI:96170 |
| *Hoxa10* | Gene |  | MGI:96180 |
| *Hoxa11* | Gene |  | MGI:96171 |
| *Hoxa13* | Gene |  | MGI:96172 |
| *Hoxa2* | Gene |  | MGI:96174 |
| *Hoxa3* | Gene |  | MGI:96175 |
| *Hoxa4* | Gene |  | MGI:96176 |
| *Hoxa5* | Gene |  | MGI:96177 |
| *Hoxa6* | Gene |  | MGI:96178 |
| *Hoxa7* | Gene |  | MGI:96179 |
| *Hoxa9* | Gene |  | MGI:96179 |
| *Hprt^tm1(CAG-mCherry/Villin)Syr^* | Allele | Targeted | MGI:5512851 |
| *Hr^hr+^* | Allele | Revertant (spontaneous) | MGI:3851709 |
| *Igs2^em2(CAG-tdTomato)Gpt^* | Allele | Endonuclease-mediated | MGI:6452470 |
| *Il2^m1^* | Allele | Spontaneous | MGI:4352594 |
| *Il4ra^tm1Fbb^* | Allele | Targeted | MGI:2657172 |
| In(7Oca2;7Sox6)100H | Chromosomal aberration | Radiation induced | MGI:2681117 |
| Is(1;11)2H | Chromosomal aberration | Radiation induced | MGI:103607 |
| Is(17;In2)1Gso | Chromosomal aberration | Radiation induced | MGI:4360978 |
| Is(In;5)1H | Chromosomal aberration | Radiation induced | MGI:103837 |
| *Itpr3^C57BL/6J^* | Allele | Not applicable | MGI:3590560 |
| *Kcna5^tm1(Kcna1)Lndn^* | Allele | Targeted | MGI:2384510 |
| *Krt12^tm1.1(KRT12*L132P)Arte^* | Allele | Targeted | MGI:5902155 |
| *Map3k4^byg^* | Allele | Chemically-induced | MGI:4361339 |
| *Mdc1^Tn(pb-ZG-s)1.1Mrc^* | Allele | Transposon induced | MGI:3719413 |
| *Mp* | Heritable Phenotypic allele | Radiation induced | MGI:1861183 |
| *Nfatc1^em2(GFP/cre)Bzsh^* | Allele | Endonuclease-mediated | MGI:5804181 |
| *Nipbl^Gt(EUCE313f02)1.1Hmgu^* | Allele | Gene trapped | MGI:5644433 |
| *Nmnat2^Tn(sb-Tyr)2172.P9KK4BOve^* | Allele | Transposon induced | MGI:5473270 |
| *Nnt^C57BL/6J^* | Allele | Spontaneous | MGI:3626282 |
| *Ogg1^m2^* | Allele | Spontaneous | MGI:6157074 |
| Pou5f1^tm1.1Scho^ | Allele | Targeted | MGI:3531175 |
| *Ppcd1* | Allele | Spontaneous | MGI:4830871 |
| Rb(1.11)2Mpl | Chromosomal aberration | Robertsonian translocation | MGI:103625 |
| *Sirt1* | Gene |  | MGI:2135607 |
| T(X;16)16H | Chromosomal aberration | Radiation induced | MGI:3032981 |
| Tc(HSA14)SC20Ktom | Chromosomal aberration | Transchromosomal insertion | MGI:5305481 |
| Tg(BEST1-rtTA,tetO-cre)1Yzl | Transgene | Transgene insertion | MGI:4398912 |
| TgTn(itol2-CAG-GFP)1Dla | Transgene | Transgenic transposon insertion | MGI:4413570 |
| TgTn(mm-DTT)1Ddra | Transgene | Transgenic transposon insertion | MGI:4421555 |
| TgTn(sb-Tyr)2172Ove | Transgene | Transgenic transposon insertion | MGI:5473269 |
| Tn(sb-SBlac)15.179039Fsp | Transposon | Transposon induced | MGI:6147354 |
| *Tomt^m1Btlr^* | Allele | Chemically-induced | MGI:3800574 |
| Ts(17^16^)65Dn | Chromosomal aberration | Trisomy | MGI:3512067 |
| *Tyk2^E775K^* | Allele | Spontaneous | MGI:3706656 |
| *Usf1^soc^* | Heritable phenotypic allele | Spontaneous | MGI:5515410 |
| *Wld^s^* | Allele | Spontaneous | MGI:1857538 |
| *zoef* | Heritable phenotypic allele | Chemically-induced | MGI:5049948 |
